# Differential Activation Patterns of fMRI in Sleep-Deprived Brain: Restoring Effects of Acupuncture

**DOI:** 10.1155/2014/465760

**Published:** 2014-06-15

**Authors:** Lei Gao, Ming Zhang, Honghan Gong, Lijun Bai, Xi-jian Dai, Youjiang Min, Fuqing Zhou

**Affiliations:** ^1^Department of Medical Imaging, The First Affiliated Hospital of Xi'an Jiaotong University, 277 West Yanta Road, Xi'an, Shaanxi Province 710061, China; ^2^Department of Radiology, The First Affiliated Hospital of Nanchang University, Nanchang, Jiangxi Province 330006, China; ^3^The Key Laboratory of Biomedical Information Engineering, Department of Biomedical Engineering, School of Life Science and Technology, Xi'an Jiaotong University, Ministry of Education, China; ^4^Acupuncture & Rehabilitation Department, Affiliated Hospital of Jiangxi University of Traditional Chinese Medicine, Nanchang, Jiangxi Province 330006, China

## Abstract

Previous studies suggested a remediation role of acupuncture in insomnia, and acupuncture also has been used in insomnia empirically and clinically. In this study, we employed fMRI to test the role of acupuncture in sleep deprivation (SD). Sixteen healthy volunteers (8 males) were recruited and scheduled for three fMRI scanning procedures, one following the individual's normal sleep and received acupuncture SP6 (NOR group) and the other two after 24 h of total SD with acupuncture on SP6 (SD group) or sham (Sham group). The sessions were counterbalanced approximately two weeks apart. Acupuncture stimuli elicited significantly different activation patterns of three groups. In NOR group, the right superior temporal lobe, left inferior parietal lobule, and left postcentral gyrus were activated; in SD group, the anterior cingulate cortex, bilateral insula, left basal ganglia, and thalamus were significantly activated while, in Sham group, the bilateral thalamus and left cerebellum were activated. Different activation patterns suggest a unique role of acupuncture on SP6 in remediation of SD. SP6 elicits greater and anatomically different activations than those of sham stimuli; that is, the salience network, a unique interoceptive autonomic circuit, may indicate the mechanism underlying acupuncture in restoring sleep deprivation.

## 1. Introduction

Acupuncture is an important element of traditional Chinese medicine (TCM) that can be traced back for at least 4,000 years. In recent years, it has gained great popularity as an alternative and complementary therapeutic intervention in the Western medicine. Neuroimaging techniques have provided new insights into the anatomy and physiological function underlying acupuncture [1–13].

Acupuncture is widely used in insomnia clinically and empirically; however, the potential neural mechanism underlying the therapeutic effects of acupuncture remains little known. As one of the most prevalent health complaints worldwide, insomnia affects approximately 10% of the population in Western industrialized countries [14] and is associated with a marked reduction in quality of life, increased fatigue, cognitive impairments, mood disturbances, and physical complaints due to its chronic sleep loss [15]. Acute sleep loss, or sleep deprivation (SD), to some extent, is an alternative form of acute insomnia. Because of its maneuverability, much research work has been carried out in short-term sleep deprivation (24 h) and found that sleep deprivation adversely affects brain function and cognitive domains [16, 17].

Many studies have suggested a remediation role of acupuncture on Sanyinjiao acupoint for sleep disturbance [18, 19]. The Sanyinjiao acupoint, also known internationally as Spleen 6 (SP6), is the junction point of the liver, spleen, and kidney meridians based on principles of TCM, and it is proposed to strengthen the spleen, resolve and expel dampness, and restore balance to the yin and blood, liver, and kidneys [20]. If acupuncture induces homeostatic force in renormalizing the neuronal responses, then activation patterns involved may be differentially affected by acupuncture or sham stimuli under conditions of SD. In the present study, we employed functional magnetic resonance imaging (fMRI) to insight the role of acupuncture on SP6 in sleep deprivation induced cortical activation. The use of fMRI to assess neuronal activity in response to acupuncture stimuli allows us to examine not only neuronal processes regulating acupuncture but also the biphasic regulation effects of acupuncture.

## 2. Materials and Methods

### 2.1. Subjects

Sixteen healthy volunteers (8 females, mean age of 22.1 ± 0.8 years) were recruited in this study after giving informed consent. Participants were selected from respondents to a web-based questionnaire. They should meet the following criteria: (1) of right hand according to the modified Edinburgh Handedness Questionnaire [21]; (2) of 20 and 24 years of age; (3) of habitual good sleeping habits (sleeping no less than 6.5 h each night for the past one month); (4) of no extreme morning or evening chromotype (score no greater than 22 on a modified Morningness-Eveningness Scale, [22]); (5) of no long-term medications; (6) of no symptoms associated with sleep disorders; (7) of no history of any psychiatric or neurologic disorders; (8) of no history of drug abuse and current use of antidepressant or hypnotic medications; (9) of acupuncture naive. Participants had an average of 15.7 ± 1.2 years of education. This study was approved by the Medical Research Ethics Committee and Institutional Review Board of The First Affiliated Hospital of Nanchang University.

### 2.2. Experimental Protocol

All subjects were scheduled for three fMRI scanning procedures, one following the individual's normal sleep and received acupuncture at SP6 (NOR group) and the other two after a night of total SD with acupuncture on SP6 (SD group) or sham (Sham group). The sessions were counterbalanced and approximately two weeks apart to minimize the residual effects of SD on cognition.

Acupuncture was performed at the acupoint SP6 on the right leg (Sanyinjiao, located in the medial lower leg, 9-10 cm above the prominence of the medial malleolus (ankle bone), and closed to the medial crest of the tibia). The needles used in the acupuncture protocol were sterile, disposable, and stainless steel acupuncture needles, which would not distort MR images, measuring 0.3 mm in diameter and 40 mm in length. The needle was inserted in SP6 with a depth of approximately 1.5 cm. Stimulation was then delivered by a balanced “tonifying and reducing” technique [1] and rotated manually clockwise and counterclockwise for 1 min at a rate of 60 times per min. Acupuncture was performed with “2 min stimuli-2 min rest-2 min stimuli” program during the task-state scanning. The procedure was performed by the same experienced and licensed acupuncturist on all subjects.

For the control of acupuncture manipulation, subjects also received the sham stimulation at a nonmeridian focus near SP6 (2-3 cm inwards from SP6) on the right leg using the same timing protocol as in the acupuncture run. The sham stimulation was delivered with the needle depth, stimulation intensity, and manipulation method identical to those used in the SP6 run.

### 2.3. fMRI Scanning Procedure

**Figure 1:**
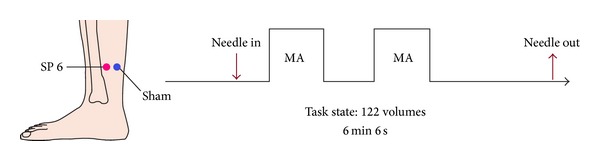
Experimental paradigm. SP6 and Sham were located on the right leg. The arrows indicated the time points of needle insertion and withdrawal. The epoch of acupuncture manipulation lasted for “2 min-MA-2* *min-rest-2* *min-MA” as shown by the framework.

Functional scanning was incorporated with three runs in each session. Two resting-state runs, each lasting 4 min, were separated by a 6 min-6 seconds task-state block-designed run (Figure 1). Resting-state data were not presented in the current study. During the scanning, subjects lay supine on the scanner bed, wearing earplugs to suppress scanner noise and with the head immobilized by cushioned supports. They were instructed to keep their eyes closed and their minds clear and remain awake. In addition, the feelings of* deqi* were collected at the end of the session, including the soreness, numbness, fullness, heaviness, and dull pain. Subjects were asked to rate each component of the* deqi* feeling they had experienced during the stimulation period using a visual analog scale (VAS). The VAS was scaled at 0 = no sensation, 1–3 = mild, 4–6 = moderate, 7-8 = strong, 9 = severe, and 10 = unbearable sensations. Because the sharp pain was considered an inadvertent noxious stimulation, we excluded the subjects from further analysis if they experienced sharp pain (greater than the mean by more than two standard deviations). Among all the participants, only one experienced the sharp pain and was removed from further analysis.

### 2.4. MRI Data Acquisition

fMRI data were collected on a SIEMENS Trio 3.0 T scanner. Each subject lied on supine with the head in neutral position fixed comfortably by a belt and foam pads during the test. The scanning sessions included (1) localizer, (2) T1 MPRAGE anatomy (176 sagittal slices, thickness/gap = 1.0/0 mm, in-plane resolution = 256 × 256, FOV (field of view) = 240 mm × 240 mm, TR (repetition time) = 1,900 ms, TE (echo time) = 2.26 ms, and flip angle = 15°), (3) EPI-BOLD (36 axial slices, echo-planar imaging pulse sequence, thickness/gap = 5.0/1 mm, in-plane resolution = 64 × 64, TR = 3,000 ms, TE = 30 ms, flip angle = 90°, and FOV = 240 mm × 240 mm).

### 2.5. fMRI Data Analysis

All preprocessing and data analyses were performed by using SPM8 (Wellcome Department of Cognitive Neurology, London, UK). For each participant, the first 2 scans of each task-state run were discarded, and the remaining images were slice-time corrected and spatially realigned to the first volume of the first run to correct for motion. The structural scan was coregistered to a mean image of the realigned functional scans. The coregistered functional scans were then normalized to the Montreal Neurological Institute template brain (resampled voxel size = 3 × 3 × 3 mm^3^) and spatially smoothed with a Gaussian kernel of 8 mm.

To investigate the acupuncture effect, general linear model (GLM) was used to analyze the block-designed data. Vectors of stimulus onsets were created for each of the acupuncture and rest conditions and convolved with the canonical hemodynamic response function. A 480 s temporal high-pass filter was applied to the data to remove low-frequency artifacts. Contrasts for acupuncture versus rest in three groups (i.e., NOR, SD, and Sham) were created for each subject. Thresholds for active brain regions were set at a cluster extent of >10 voxels and a voxel level of *P* < 0.001. After individual analyses, a one way within-subject ANOVA and post hoc were performed and paired *t*-test for group analysis was performed by using the same statistical parameters to compare regional brain activity with acupuncture versus rest for rested wakeful and sleep deprivation. Statistical analyses were performed by using SPM8. Only the coordinates from the largest cluster for each brain region are presented in the main tables for regions with multiple locations.

## 3. Results

4 subjects were excluded on discovery of excessive head motion or experienced the sharp pain during the task. A total of 12 participants (5 men) completed the fMRI protocol.

**Figure 2:**
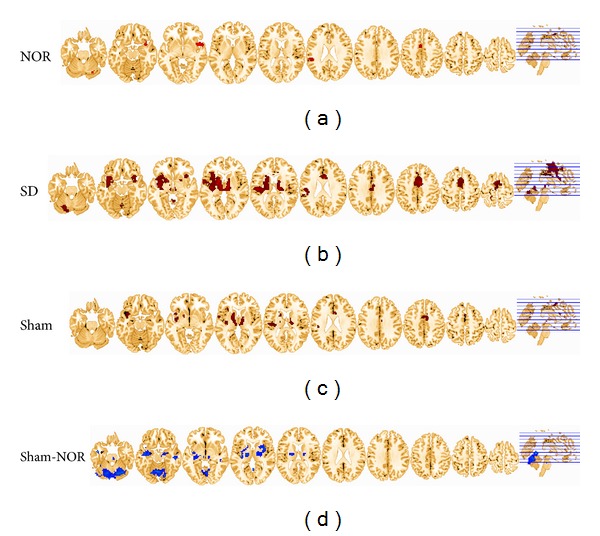
(a) Activations for NOR group during acupuncturing on SP6, compared with a resting baseline are shown. (b) Activations for SD group during acupuncturing on SP6, compared with a resting baseline are shown. (c) Activations for Sham group during acupuncturing on Sham, compared with a resting baseline are shown. (d) Group differences between Sham and SD. Cool color indicates that the Sham group had decreased activations compared with the SD group. All images were normalized to the standardized space defined by MNI using the structural MRI of each subject.

*Rested-Wakeful Condition: Acupuncture versus Baseline.* Under habitual sleep, responses to acupuncture versus baseline stimuli were found in left middle frontal area (MFA), medial frontal gyrus (MFG), precentral area (PreCG), postcentral area (PoCG), left putamen (PUT), anterior cingulate (ACC), right superior temporal gyrus (STG), insula (INS), and right inferior parietal lobe (IPL) (Figure 2(a), Table 1).

*Sleep Deprivation Condition: Acupuncture versus Baseline.* Under the sleep deprivation condition, greater neuronal activation was observed in the responses to acupuncture versus baseline. Activations were found in the right ACC, bilateral thalamus, bilateral INS, right MFG, bilateral STG, bilateral middle temporal gyrus (MTG), left PoCG, bilateral caudate (CAU), right uncal gyrus, left PUT, fusiform, right cerebellum anterior lobe, and so forth (Figure 2(b), Table 2).

*Sleep Deprivation Condition: Sham versus Baseline.* Under the sleep deprivation condition, Sham induced activations in the left superior frontal gyrus (SFG), bilateral MFG, bilateral PreCG, bilateral thalamus, bilateral INS, left pons, and left cerebellum posterior lobe (Figure 2(c), Table 3).

*Sleep Deprivation Condition: Sham versus Acupuncture.* To investigate the differences between Sham HAM and SP6 in sleep-deprived condition, a paired *t*-test was performed between SD and Sham groups. Results indicated that the group differences of activations were significantly decreased in the Sham group than that of in the SD group, including right INS/thalamus, bilateral MTG, right hippocampus, and left cerebellum (Figure 2(d); Table 4).

## 4. Discussion

The present study investigated the activation patterns of acupuncture in SP6 in different sleep conditions. We found that acupuncture in SP6 increased regional brain activity primarily in the ACC, INS, basal ganglia, and limbic system after sleep deprivation, while Sham induced activations in the left SFG, bilateral MFG, bilateral precentral area, bilateral thalamus, bilateral INS, left pons, and left posterior lobe of the cerebellum. Although acupuncture also elicited increases the regional brain activity in the MFC/ACC, insular, and IPL during rested wakeful, both the extent and intensity of activation were reduced and much less widespread. Our findings may suggest that sleep deprivation alters neuronal activity, which predisposes individuals to contraction and enhanced responses to acupuncture and may partly explain the biphasic regulation effects of acupuncture.

Sleep constitutes an approximate one-third of the human lifetime, and many hypotheses have been proposed about its role in physiological functions, including homeostatic restoration, thermoregulation, tissue repair, immune control, memory processing, and consolidation [23, 24]. Sleep deprivation has been shown to have a negative impact on the brain and health [17]. Sleep deprivation falls under the category of “fatigue” and “sleepless” in TCM and stands for “excessive lassitude,” “visceral dysfunction,” deficiency of “qi and blood,” and yin-yang disharmony, though not yet been severe [25]. The remediation is to re-establish the equilibrium between them. Applying pressure at this acupuncture meridian can refresh the mind, sedate, nourish spleen and stomach, nourish liver, and produce other health effects [26].

In the present study, we found different neuronal activity patterns evoked by acupuncture under sleep deprivation and rested wakeful. In NOR group, a state of physical fitness, SP6 acupuncture evoked activations may indirectly reflect the neuronal responses of these regions, including MPFC, insula, putamen, lateral parietal lobes, and sensorimotor areas, which are frequently recruited in executive-control, sensory information processing, visceral regulation, social emotion, and self-awareness. Activations in these regions may be consistent with its widespread functions of SP6 in mental [27], gynecological [20, 26], and neurological diseases [27] as recorded.

In the SD group, more widespread brain regions were activated and the activation level as well as the strength was significantly higher than that of NOR and Sham group. Besides the above-mentioned activations in the NOR, ACC and insula were especially significantly activated both in extent and intensity. A prominent cognitive role of the ACC is processing errors and conflict [28, 29]. Salience network, composed of the anterior insular cortex and ACC, has received increasing attention [30–32]. This brain network is supposed to be implicated in multiple functions, ranging from attention to interoception and subjective awareness [33, 34]. The salience network integrates external sensory stimuli with internal states, and the anterior insula acts as a hub, mediating interactions between large-scale networks involved in externally and internally oriented cognitive processing [34, 35]. Most remaining nodes in the salience network are subcortical sites for emotion, homeostatic regulation, and reward [34]. Regions such as the lateral prefrontal cortex (PFC) and lateral parietal cortex are consistently recruited by cognitively demanding tasks and are critical for guidance of thought and behavior [36, 37]. In sleep deprivation, SP6 may exert an everlasting influence over the short-time period, as well as less maladaptive stimulation.

In Sham group, significant activations in the thalamus, pons, and basal ganglia were involved. This may indicate the impact of activations on the sleep deprivation itself. Under the extreme sleepy condition, more brain regions were involved in the compensation of maintaining the awareness and alert (thalamus and pons), while deactivations that occurred in the regions support advanced cognitive functions, dorsolateral prefrontal cortex, anterior cingulate cortex, and parietal lobes. It is generally believed that the Sham mainly relates to the processes of maladaptive stimulation [1, 2]. But much of the effects represent sleep deprivation, emotional and visceral processing—the left medial prefrontal. Naturally, we would suppose that low activation level of brain salience network in response to salient Sham stimuli could be explained as a failure in remediation, because such response would indicate that more stimuli are necessary to produce salient stimuli in Sham.

****Interestingly, in the first individual level analysis the extent of activations in NOR was greater than SD and Sham group, and second level group statistics vice versa, that is, a relatively weak group effect in NOR and greater group effects both in SD and Sham. We speculated that the reasons for this inconsistent were likely to reflect the biphasic regulation effects of acupuncture. In the NOR group, acupuncture in SP6 reveals sparse results which may relate to its multiple functions. In sleep deprivation, an imbalance occurs; acupuncture stands for a homeostatic force to renormalize the yin and yang, biphasic regulation effects of acupuncture. Another study also reported similarities. Our results support this hypothesis that the effects of acupuncture were closing to launch homeostatic regulation [9, 38].

## 5. Limitations

There are several limitations in this study. First, in a relatively small sample in our study, the results for group comparison were not corrected for multiple comparisons; therefore, they should only be considered an exploratory analysis. Second, some flaws exist in our protocol, for example, a lack of sham stimuli in rested wakeful condition. The remediation of acupuncture in sleep deprivation cannot be totally inferred. Third, block design permits the observation of an immediate acupuncture effect rather than its post effects which are more valuable clinically. The mechanism still need to be further evaluated.

## 6. Conclusion

Different activation patterns suggest an important role of acupuncture on SP6 in remediation of SD. SP6 elicits greater and anatomically different activations than the same stimuli, that is, the salience network. A unique interoceptive autonomic circuit may, partly, indicate the mechanism underlying acupuncture in restoring sleep deprivation.

## Figures and Tables

**Table 1:** Activations for NOR group during acupuncturing on SP6 compared with a resting baseline are shown. (*P* < 0.001, cluster >10 voxels, uncorrected).

Brain regions	MNI coordinates (*x*, *y*, *z*)			BA	L/R	Voxels	*T* values
Middle frontal gyrus	−36	47	22	10	L	25	4.889
ACC	−3	2	46	32/24	L	22	4.9336
Superior temporal gyrus	54	8	−2	22/38	R	115	6.738
Inferior parietal lobule	−57	−34	25	40	R	50	4.928
Precentral gyrus	48	0	9	44	R	20	8.8206
Postcentral gyrus	−48	−16	22	3	L	25	5.022
Thalamus	3	−9	6	—	R	16	6.115
Putamen	−21	13	4	—	L	20	5.8152
Cerebellum	30	−70	−23	—	R	47	6.2835

**Table 2:** Activations for SD group during acupuncturing on SP6 compared with a resting baseline are shown (*P* < 0.001, cluster >10 voxels, uncorrected).

Brain regions	MNI coordinates (*x*, *y*, *z*)			BA	L/R	Voxels	*T* values
Inferior frontal gyrus	33	11	−14	13	R	74	5.908
Inferior/medial frontal gyrus	−34	13	−4	13/47	L	180	6.1011
Superior temporal gyrus	57	5	1	22	R	58	4.5028
Superior temporal gyrus	−40	8	−14	38	L	26	5.01
Fusiform	−15	8	−2	22/38	L	2060	10.1924
ACC	9	−1	49	24/6/8	R	720	7.608
ACC	6	14	25	24/33	R	47	7.1919
Insula	42	−16	13	13	R	69	4.6696
				13	L	366	
Postcentral gyrus	−15	−40	70	3	L	24	5.1276
Thalamus	3	−9	6	—	R	157	7.003
					L	131	—
Caudate	16	−13	20	—	L	54	5.542
Caudate	−14	−13	19		R	67	—
Cerebellum	−18	−67	−29	—	L	194	5.9275
Cerebellum	39	−55	−35		R	59	4.5446
Cerebellum	3	−52	−2		R	71	5.6171

**Table 3:** Activations for Sham group during acupuncturing on SP6, compared with a resting baseline are shown (*P* < 0.001, cluster >20 voxels, uncorrected).

Brain regions	MNI coordinates (*x*, *y*, *z*)			BA	L/R	Voxels	*T* values
Medial frontal gyrus	12	3	54	6/24	R	31	6.2621
Medial frontal gyrus	−12	−18	60	6/3/4	L	285	8.7196
Thalamus	3	−9	6	—	R	146	8.1692
				—	L	210	
Pon	−18	−24	−33	—	L	20	7.5021
Insula	42	12	−9	13	R	20	4.6318
Putamen	−21	6	−12	—	L	12	5.4318
Postcentral gyrus	−48	−16	22	3	L	25	5.022
Cerebellum	−30	−66	−21	—	L	102	6.1046
Cerebellum	−33	−51	−48	—	L	72	5.6012

**Table 4:** Group differences between Sham and SD are shown (*P* < 0.001, cluster >20 voxels, uncorrected).

Brain regions	MNI coordinates (*x*, *y*, *z*)			BA	L/R	Voxels	*T* values
Insula/thalamus	33	21	3	13	R	165	−6.3468
Middle temporal gyrus	48	−24	−12	21	R	63	−8.3397
Middle temporal gyrus	−45	−3	−24	21/38/13	L	423	−9.6562
Hippocampus	21	−9	−12	—	R	48	−6.3265
Cerebellum	0	−57	3	—	L	963	−8.3057

## References

[1] Bai L, Qin W, Tian J (2009). Time-varied characteristics of acupuncture effects in fMRI studies. *Human Brain Mapping*.

[2] Bai L, Qin W, Tian J (2009). Acupuncture modulates spontaneous activities in the anticorrelated resting brain networks. *Brain Research*.

[3] Liu P, Qin W, Zhang Y (2009). Combining spatial and temporal information to explore function-guide action of acupuncture using fMRI. *Journal of Magnetic Resonance Imaging*.

[4] Bai L, Yan H, Li N (2010). Neural specificity of acupuncture stimulation at pericardium 6: evidence from an fMRI study. *Journal of Magnetic Resonance Imaging*.

[5] Li L, Qin W, Bai L, Tian J (2010). Exploring vision-related acupuncture point specificity with multivoxel pattern analysis. *Magnetic Resonance Imaging*.

[6] Bai L, Tian J, Zhong C (2010). Acupuncture modulates temporal neural responses in wide brain networks: evidence from fMRI study. *Molecular Pain*.

[7] Zhang Y, Li K, Ren Y (2014). Acupuncture modulates the functional connectivity of the default mode network in stroke patients. *Evidence-Based Complementary and Alternative Medicine*.

[8] Xie Z, Cui F, Zou Y, Bai L (2014). Acupuncture enhances effective connectivity between cerebellum and primary sensorimotor cortex in patients with stable recovery stroke. *Evidence-Based Complementary and Alternative Medicine*.

[9] Bai L, Lao L (2013). Neurobiological foundations of acupuncture: the relevance and future prospect based on neuroimaging evidence. *Evidence-Based Complementary and Alternative Medicine*.

[10] Wei W, Bai L, Wang J (2013). A longitudinal study of hand motor recovery after sub-acute stroke: a study combined FMRI with diffusion tensor imaging. *PLoS ONE*.

[11] You Y, Bai L, Dai R (2013). Altered hub configurations within default mode network following acupuncture at ST36: a multimodal investigation combining fMRI and MEG. *PLoS ONE*.

[12] Feng Y, Bai L, Ren Y (2012). FMRI connectivity analysis of acupuncture effects on the whole brain network in mild cognitive impairment patients. *Magnetic Resonance Imaging*.

[13] Qin W, Bai L, Dai J (2011). The temporal-spatial encoding of acupuncture effects in the brain. *Molecular Pain*.

[14] Ohayon MM (2002). Epidemiology of insomnia: what we know and what we still need to learn. *Sleep Medicine Reviews*.

[15] Morin CM, Benca R (2012). Chronic insomnia. *The Lancet*.

[16] Basner M, Rao H, Goel N, Dinges DF (2013). Sleep deprivation and neurobehavioral dynamics. *Current Opinion in Neurobiology*.

[17] Goel N, Rao H, Durmer JS, Dinges DF (2009). Neurocognitive consequences of sleep deprivation. *Seminars in Neurology*.

[18] Sok SR, Erlen JA, Kim KB (2003). Effects of acupuncture therapy on insomnia. *Journal of Advanced Nursing*.

[19] Scheid V (2007). Traditional Chinese medicine—what are we investigating?. The case of menopause. *Complementary Therapies in Medicine*.

[20] Kashanian M, Shahali S (2010). Effects of acupressure at the Sanyinjiao point (SP6) on the process of active phase of labor in nulliparas women. *Journal of Maternal-Fetal and Neonatal Medicine*.

[21] Oldfield RC (1971). The assessment and analysis of handedness: the Edinburgh inventory. *Neuropsychologia*.

[22] Horne JA, Ostberg O (1976). A self-assessment questionnaire to determine morningness-eveningness in human circadian rhythms. *International Journal of Chronobiology*.

[23] Tononi G, Cirelli C (2006). Sleep function and synaptic homeostasis. *Sleep Medicine Reviews*.

[24] Abel T, Havekes R, Saletin JM, Walker MP (2013). Sleep, plasticity and memory from molecules to whole-brain networks. *Current Biology*.

[25] Cao H, Pan X, Li H, Liu J (2009). Acupuncture for treatment of insomnia: a systematic review of randomized Controlled trials. *Journal of Alternative and Complementary Medicine*.

[26] Chen H, Chen C (2004). Effects of acupressure at the Sanyinjiao point on primary dysmenorrhoea. *Journal of Advanced Nursing*.

[27] Lin Y (1995). Acupuncture treatment for insomnia and acupuncture analgesia. *Psychiatry and Clinical Neurosciences*.

[28] Eisenberger NI, Lieberman MD, Williams KD (2003). Does rejection hurt? An fMRI study of social exclusion. *Science*.

[29] Shenhav A, Botvinick M, Cohen J (2013). The expected value of control: an integrative theory of anterior cingulate cortex function. *Neuron*.

[30] Uddin LQ, Supekar K, Lynch CJ (2013). Salience network-based classification and prediction of symptom severity in children with autism. *JAMA Psychiatry*.

[31] Seeley WW, Menon V, Schatzberg AF (2007). Dissociable intrinsic connectivity networks for salience processing and executive control. *The Journal of Neuroscience*.

[32] Chiong W, Wilson SM, D’Esposito M (2013). The salience network causally influences default mode network activity during moral reasoning. *Brain*.

[33] Uddin LQ, Supekar K, Lynch CJ (2013). Salience network-based classification and prediction of symptom severity in children with autism. *JAMA Psychiatry*.

[34] Seeley WW, Menon V, Schatzberg AF (2007). Dissociable intrinsic connectivity networks for salience processing and executive control. *The Journal of Neuroscience*.

[35] Chen AC, Oathes DJ, Chang C (2013). Causal interactions between fronto-parietal central executive and default-mode networks in humans. *Proceedings of the National Academy of Sciences of the United States of America*.

[36] Ramnani N, Owen AM (2004). Anterior prefrontal cortex: insights into function from anatomy and neuroimaging. *Nature Reviews Neuroscience*.

[37] Gottlieb J (2007). From thought to action: the parietal cortex as a bridge between perception, action, and cognition. *Neuron*.

[38] Dhond RP, Kettner N, Napadow V (2007). Neuroimaging acupuncture effects in the human brain. *Journal of Alternative and Complementary Medicine*.

